# Ginsenoside Rb1 Interfered with Macrophage Activation by Activating PPARγ to Inhibit Insulin Resistance in Obesity

**DOI:** 10.3390/molecules28073083

**Published:** 2023-03-30

**Authors:** Hongyue Ding, Jinxiang Dong, Yuqi Wang, Qiang Huang, Jie Xu, Zhidong Qiu, Fan Yao

**Affiliations:** School of Pharmaceutical Sciences, Changchun University of Chinese Medicine, Changchun 130117, China

**Keywords:** ginsenoside Rb1, insulin resistance, inflammation, PPARγ, network pharmacology

## Abstract

Type 2 diabetes (T2D) is characterized by insulin resistance (IR), often accompanied by inflammation. Macrophage activation acts as an inflammatory response, which is characterized by macrophage recruitment in the initial stage. Ginsenoside Rb1 (Rb1) is a main active ingredient, which is known for its fat-reducing, anti-inflammatory effects. To clarify that Rb1 regulates macrophage activation in adipose tissue and improves tissue inflammation, network pharmacology and molecular docking were used for target prediction and preliminary validation. By constructing the co-culture model of adipose-derived stem cells (ADSC) and primary macrophage (PM), the body adipose tissue microenvironment was simulated to observe the adipogenesis degree of adipocytes under the effect of Rb1. The levels of cytokines, macrophage polarization, and protein or RNA expression in the inflammatory signaling pathway were finally detected. The results showed that 89 common targets of T2D-Rb1 were obtained after their intersection. Furthermore, according to the results of the KEGG pathway and PPI analysis, PTGS2 (COX-2) is the downstream protein of PPARγ-NF-κB. The molecular binding energy of PPARγ-Rb1 is −6.8 kcal/mol. Rb1 significantly inhibited the increase in MCP-1, TNF-α, and IL-1β induced by hypertrophic adipocytes supernatant and promoted the expression of IL-10. Rb1 inhibited the activation of inflammatory macrophages and PM migration and upregulated PPARγ expression with the blocking of NF-κB activation. Additionally, Rb1 promoted the expression of IRS1 and PI3K in the insulin signal pathway, which had a similar effect with ROS. Therefore, Rb1 might affect macrophage activation through PPARγ, which might alleviate obese insulin resistance in T2D early stage.

## 1. Introduction

Type 2 diabetes mellitus (T2D) is a common chronic metabolic disease, and insulin resistance (IR) is its main feature [1]. In recent years, the prevalence of overweight and obesity has been increasing year by year, and it has become one of the major diseases [2]. However, excessive intake of high fat and high sugar has been considered a potential factor in the increased incidence of T2D [3]. Obesity is a pathological phenomenon in which lipids accumulate in the body and is also the main factor inducing IR. Adipose tissue (AT) is generally regarded as a lipid storage organ, and visceral adipose tissue plays a more important role in endocrine regulation [4]. Adipose tissue regions usually contain mature adipocytes, stem cells, other stromal cells, and reactive immune cells, such as macrophages [5]. Macrophages are immune cells that, in order to maintain homeostasis, conduct chemotactic directional movement, moving along the concentration gradient of certain chemicals and aggregating to the lesion site where these substances are released. When adipose tissue homeostasis is disrupted, macrophages rapidly increase from about 10% to 50% of adipose tissue cells, which is also one of the main manifestations of a regional immune response in visceral adipose tissue, namely, macrophage activation [6].

The activation of macrophages is mainly based on the different cell phenotypes and secreted cytokines. Macrophages can be divided into two types, the classical activation pathway M1 type and the selective activation pathway M2 type [7]. The main function of M1 macrophages is to promote the secretion of early proinflammatory cytokines, such as TNF-α, IL-1β, iNOS, and MCP-1 [8]. The main function of M2 macrophages is to inhibit the inflammatory response and alleviate tissue damage. Two important nuclear transcription factors, PPARγ and NF-κB, are also key targets in the process of macrophage polarization regulation [9,10,11]. In addition to promoting adipogenesis, PPARγ also plays a regulatory role in macrophage activation. Studies have shown that oral administration of Rb1 in mice can reduce body weight and improve glucose and lipid metabolism by upregulating PPARγ and AQP7 protein levels [12]. Therefore, PPARγ can affect the secretion process of inflammatory cells in the early stage of macrophage activation and may form a feedback regulatory pathway.

Ginsenoside Rb1 is the main component of ginsenoside [13]. Researchers found that Rb1 has a lipid-lowering effect, reduces adiponectin secretion in adipocytes 3T3-L1, increases insulin sensitivity, and increases AMPK phosphorylation in WT mouse liver [14]. In addition, it has been found that Rb1 can improve metabolic disorders in high-fat diet-induced obese mice related to gut microbiota regulation, and oral administration of Rb1 significantly reduces serum LDL-c, TG, insulin, and insulin resistance index (HOMA-IR) in high-fat diet (HFD) mice. Rb1 can improve the relative abundance of key bacterial genera in the gastrointestinal tract and, thus, improve obesity, which is conducive to type 2 diabetes [15]. Previous studies have shown that Rb1 may partially ameliorate obesity through the MSTN/FNDC5 signaling pathway [16].

Therefore, in order to further prove that Rb1 can relieve T2D, we focused on this study of Rb1 network pharmacology study and explored its possible mechanism of T2D effect or T2D early prevention. Then by building molecular docking, ADSC fat cells, and the original generation PM co-culture model, a more realistic simulation of fat cells under the microenvironment would demonstrate that ginsenoside Rb1 could activate macrophages and alleviate insulin resistance in T2D early stage.

## 2. Results

### 2.1. The Obtainment of Potential Targets

Target prediction of Rb1 was performed through the PubChem database and the SwissTargetPrediction database. After screening the results, a total of 100 potential targets of Rb1 active ingredients were obtained.

### 2.2. Acquisition of Common Targets for T2D-Rb1

**Figure 1:**
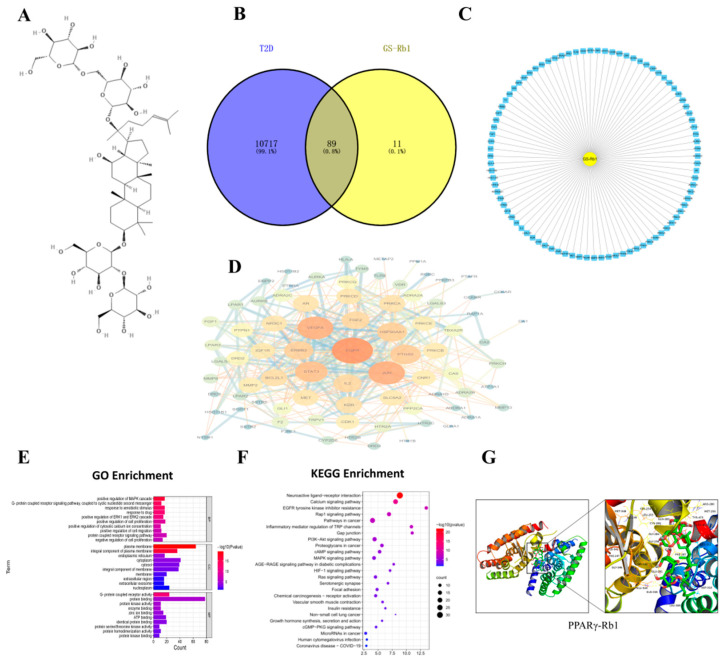
(**A**) The structure of ginsenoside Rb1. (**B**) Venn diagram of component target-disease target. (**C**) Composition-common target network diagram. (**D**) PPI network of targets. (**E**) GO Enrichment. (**F**) KEGG Enrichment. (**G**) Schematic diagram of molecular docking results of PPARγ-Rb1.

Through GeneCards, OMIM, DisGeNET, CTD, and Drugbank databases, T2D targets were retrieved and collected. The results of five database targets were summarized, the duplicate genes were removed, and 10805 T2D-related targets were obtained. The targets of active components of T2D and Rb1 were input into the Venny 2.1.0 software to draw the Venny diagram, and 89 common targets of T2D-Rb1 were obtained after their intersection, as shown in Figure 1B,C.

### 2.3. PPI Network Analysis and Key Target Acquisition

Protein-protein interaction analysis of the common targets of Rb1-T2D was performed by STRING database, and the PPI network diagram was constructed by the Cytoscape 3.9.0 software, as shown in Figure 1D, where the larger the degree value (degree), the darker the node color. PTGS2 is a critical protein and a downstream protein of NF- κB- IKBα.

### 2.4. GO and KEGG Pathway Enrichment Analysis

The DAVID 6.8 database was used for biological enrichment analysis of intersection targets, and 449 GO functional enrichment items with statistical significance were obtained, including 324 biological process (BP) items, 324 molecular function (MF) items, and 64 cellular components (CC). The GO-enriched items were sorted according to P, and a smaller P indicated a higher degree of enrichment. The top 10 items in BP, CC, and MF were selected to draw a bar graph. BP, CC, and MF are mainly enriched in positive regulation of cell migration; plasma membrane, cytoplasm, and cytosol; G-protein coupled receptor activity, as shown in Figure 1E. The KEGG pathway enrichment results showed that 89 intersection targets were involved in a total of 80 pathways and the bubble map of the top; the KEGG pathways were drawn by P-ranking, as shown in Figure 1F. The pathway name, the X-axis represents the number of genes, the bubble area represents the number of pathway-enriched genes, the bubble color represents the size of the *p* value, and the bluer the bubble color indicates the higher significance of the signaling pathway. The enrichment results showed that insulin resistance is the common pathway.

### 2.5. Molecular Modeling of PPARγ-Rb1

It is generally believed that when the conformation of ligand and receptor is stable, the lower the energy, the more likely the interaction will occur. The molecular docking results showed that the binding energy of PPARγ-Rb1 was −6.8 kcal/mol, which can be viewed as shown in Figure 1G.

### 2.6. Identification of ADSC and PM

**Figure 2:**
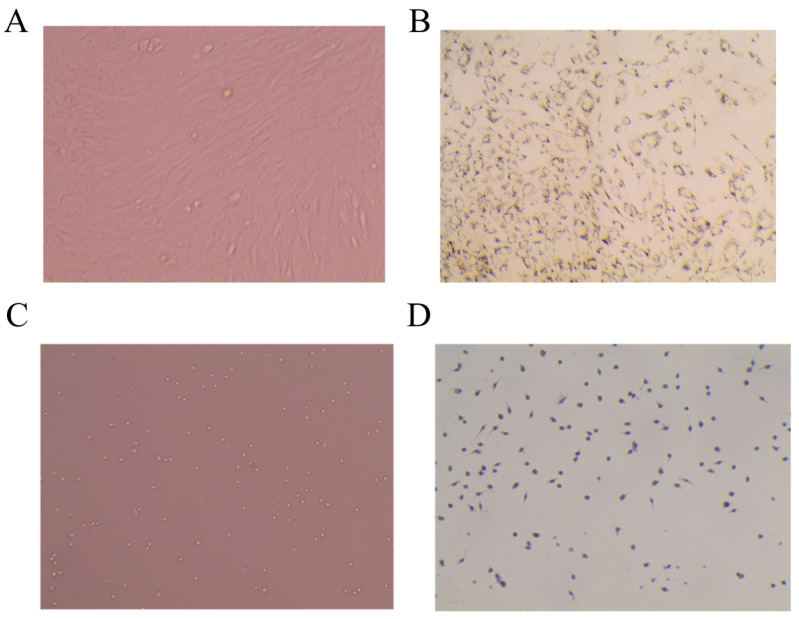
Morphological observation of adipose stem cells and peritoneal macrophages in primary culture mice. (**A**) ADSC (20×). (**B**) ADSC, oil red O staining (20×). (**C**) PM (20×). (**D**) PM-immunohistochemical CD68 staining (20×).

The ADSC cell morphology of the first passaged cells 24 h after adherent was shown in Figure 2A, showing a long spindle shape with a small number of cells appearing in lipid droplets. The third-generation cells were induced to differentiate and stained with oil red O. The results are shown in Figure 2B. The cells were induced to differentiate from long spindle shape to round shape, and the lipid droplets could be stained red after oil red O staining. Moreover, PM cell morphology is shown in Figure 2C; the cells can be seen as round, oval, or irregular, and some cells have pseudopodia. After the PMs were stained with immunohistochemical CD68, the results are shown in Figure 2D. The cultured macrophages were brown after staining, indicating that the cultured cells were macrophages.

### 2.7. Cell Viability Assay

**Figure 3:**
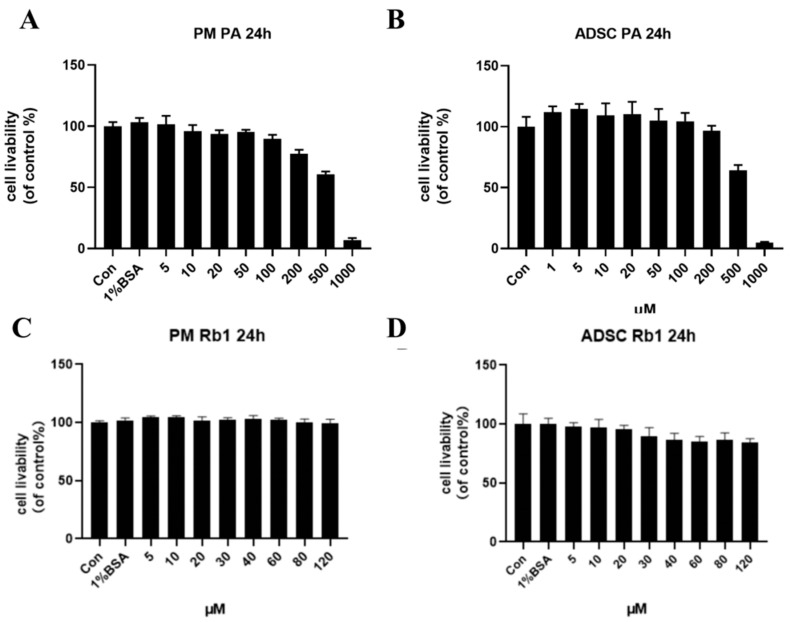
Effects of PA at different concentrations for 24 h on the cell livability of PM (**A**) and ADSC (**B**) cells; effects of Rb1 at different concentrations for 24 h on the cell livability of PM (**C**) and ADSC (**D**) cells.

Different concentrations of palmitic acid (PA) affect the viability of ADSC and PM cells. CCK8 was used to screen out an appropriate concentration for modeling. As shown in Figure 3A, 1% BSA palmitic acid solution was non-toxic to PM cells, and the survival rate of macrophages with 100 μm and 200 μm PA solution was 92.16% and 81.8%, respectively. For ADSC cells, as shown in Figure 3B, the cell livability of ADSC cells decreased to 65.8% after 500 μM PA solution. PM adherents for 24 h were treated with different concentrations of Rb1, and as shown in Figure 3C, 1% BSA had no effect on PM, and cell livability was all above 90%. ADSC adherents for 24 h were treated with different concentrations of Rb1, as shown in Figure 3D, 1% BSA had no effect on ADSC, and the cell livability could reach more than 90%. The cell livability of ADSC was 89.92% and 86.68% when Rb1 was 30 μm and 40 μm, respectively. In order to establish a suitable model of mast cells induced by PA, 100 μm concentration of PA was selected as the appropriate model concentration, and the appropriate concentration of Rb1 should be 30 μm or less.

### 2.8. Establishment of Hypertrophy Model of Mature ADSC Cells Stimulated by PA

**Figure 4:**
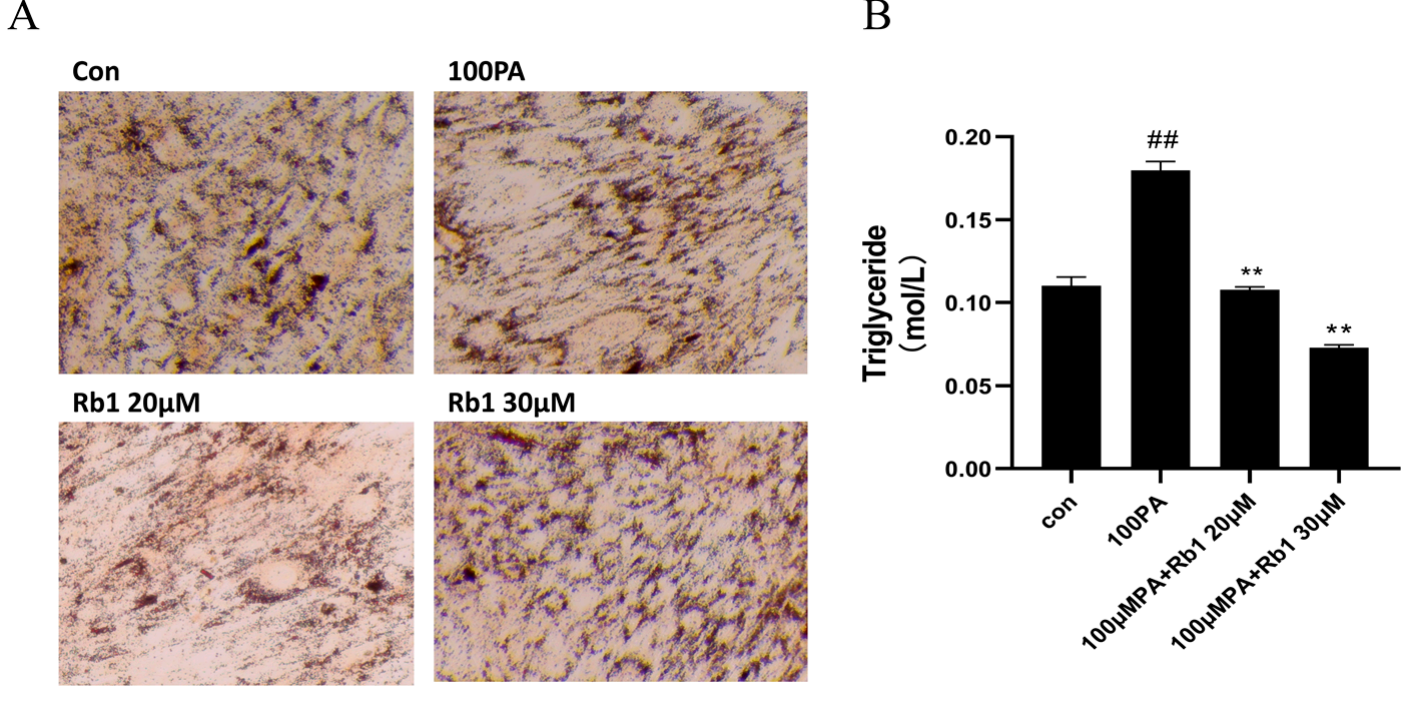
ADSC cell morphology (20×) (**A**) and triglyceride content in cells (**B**) at different concentrations (## *p* < 0.01 significantly compared with Con group; ** *p* < 0.01 significantly compared with 100 PA group).

In order to verify the establishment of the ADSC mast cell model and the improvement effect of Rb1 on the model, the differentiated ADSC cells were stimulated with PA to construct their adipocyte hypertrophy model. As shown in Figure 4A, the Con group consisted of mature ADSC cells after induced differentiation. The lipid droplets in the cells were stained red after oil Red O staining, and the mature adipocytes secreting ring lipid droplets were differentiated from the long spindle-shaped adipocytes. After stimulation with 100 μM PA, the mature ADSC cells were induced to hypertrophic adipocytes and the lipid droplets, as shown in Figure 4A,B. The cell size and triglyceride content were significantly higher than the Con group (*p* < 0.01), but after the Rb1 treatment, the cell volume became smaller, and the triglyceride content significantly decreased compared with the model group (*p* < 0.01).

### 2.9. Obesity-Induced Insulin Resistance Promotes Macrophage Polarization

**Figure 5:**
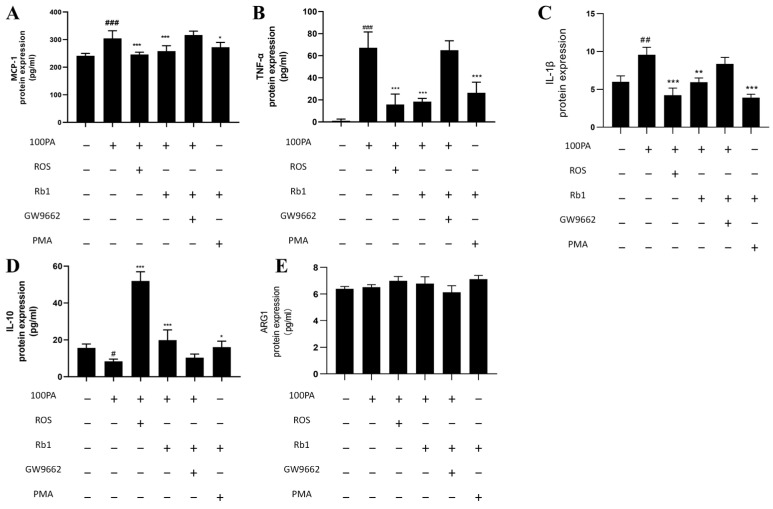
Changes in cytokine levels. (**A**) MCP-1. (**B**) TNF-α. (**C**) IL-1β. (**D**) IL-10. (**E**) ARG1. Con: control group; model: mature adipocyte supernatant was incubated with 100 μM palmitic acid. ### *p* < 0.001 significantly compared with Con group; ## *p* < 0.01 significantly compared with Con group; # *p* < 0.05 significantly compared with Con group; *** *p* < 0.001 significantly compared with model group; ** *p* < 0.01 significantly compared with model group; * *p* < 0.05 significantly compared with model group.

We investigated whether obesity-induced insulin resistance at the cellular level might affect macrophage polarization and changes in cytokine levels. The contents of M1 cytokines TNF-α and IL-1β, chemokine MCP-1, and M2 cytokines IL-10 and ARG1 in the supernatant of co-cultured ADSC cells were detected by ELISA kit. As shown in Figure 5, compared with the Con group, the model group significantly increased the displacement factor MCP-1, M1 cytokine TNF-α, IL-1β, and decreased M2 cytokine IL-10; Rosiglitazone (ROS) as a PPARγ agonist can decrease M1 and increase M2 cytokines. Rb1 and ROS act in the same direction to decrease M1 and increase M2 cytokines. GW9662 is a PPARγ inhibitor, which can counteract the effect of Rb1. PMA is an NF-κB agonist, and PMA + Rb1 can exert the same regulatory effect as Rb1.

### 2.10. Effect of Obesity-Induced Insulin Resistance on Macrophage Activation

**Figure 6:**
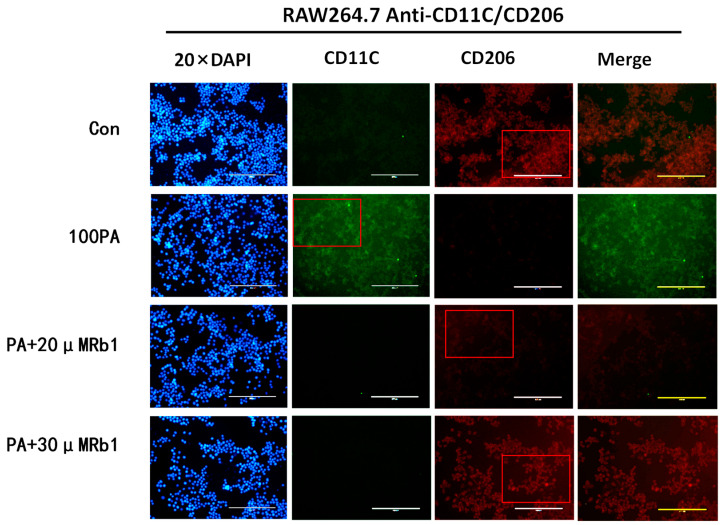
The expression of M1 and M2 types (20×). Con: control group; 100 PA: mature adipocyte supernatant was incubated with 100 μM PA; 100 PA + 10 μM Rb1 and 20 μM: mature adipocyte supernatant and ginsenoside Rb1 were incubated with 100 μM PA + 10 μM Rb1 and 20 μM Rb1.

In the process of insulin resistance caused by obesity, macrophages are not only recruited and infiltrated into adipose tissue but also accompanied by macrophage polarization. CD11C for inflammatory antibody, namely M1 macrophage surface recognition, decoupling FITC shows green fluorescent; CD206 for anti-inflammatory, namely M2 macrophage surface to identify antibody, decoupling PE shows red fluorescence. So for the study of the cellular level under the local area of adipose tissue macrophage polarization, the immunofluorescence method was used to detect the changes in the macrophage polarization process. As shown in Figure 6, macrophages in the model group showed strong green fluorescence; the intensity of red fluorescence was weakened, and the number of macrophages was less than that of the blank control group. The green fluorescence intensity of the ginsenoside Rb1 group was weaker than that of the model group, and the red fluorescence signal intensity was increased.

### 2.11. Obesity-Induced Insulin Resistance Promotes Macrophage Migration

**Figure 7:**
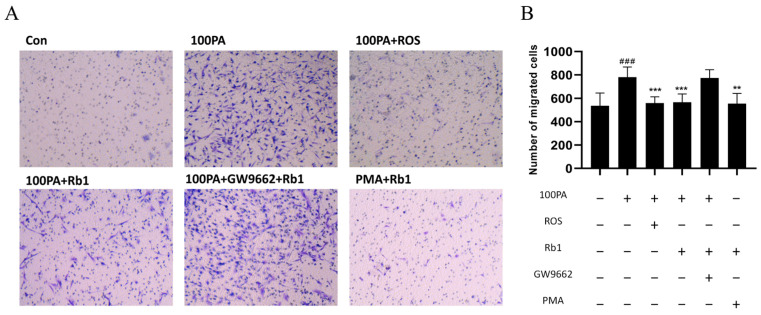
PM cells were co-cultured in the Transwell chamber and Rb1 affected the migration efficiency. (**A**) The analysis of cell migration number of view after crystal violet staining (20×) and their quantification diagram (**B**). Con: control group; model: mature adipocyte supernatant was incubated with 100 μM palmitic acid. ### *p* < 0.001 significantly compared with Con group; *** *p* < 0.001 significantly compared with model group; ** *p* < 0.01 significantly compared with model group.

The co-culture model of ADSC and PM was constructed, and the migration rate of upper macrophages was observed by crystal violet staining. The migrating cells stained with crystal violet were shown in Figure 7A, while the migration of macrophages was significantly promoted in the model group. ROS or Rb1 can reduce macrophage migration of the model group. After treatment with GW9662 4 h in advance and application of Rb1 for 24 h, the effect of Rb1 could be canceled. PMA + Rb1 group was found to inhibit macrophage migration.

### 2.12. Signaling Pathway of Rb1 Alleviating Insulin Resistance

**Figure 8:**
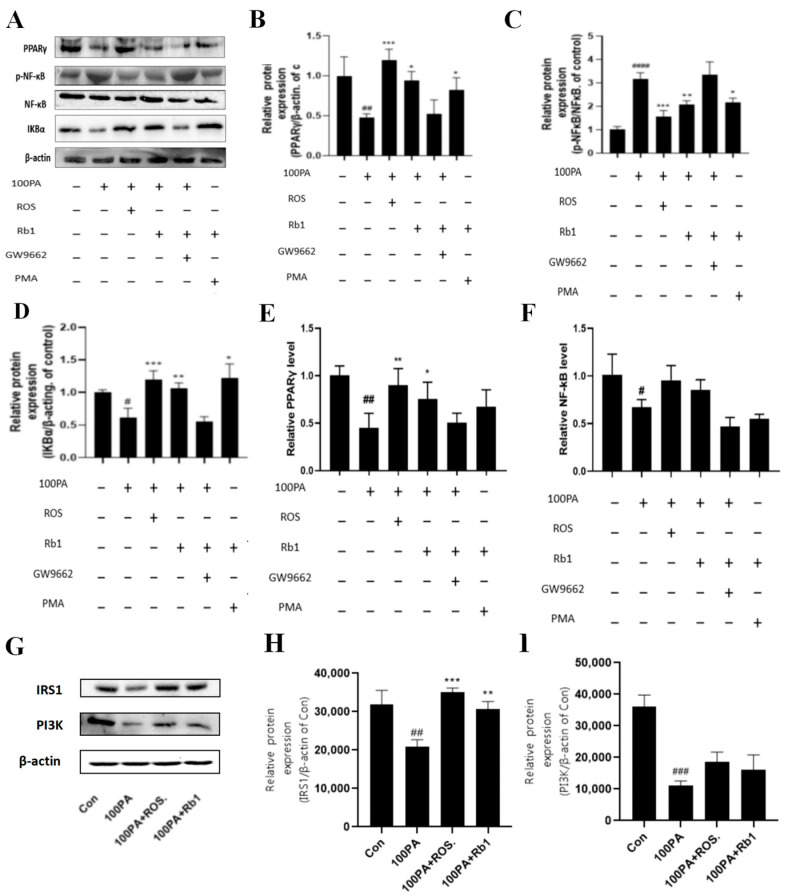
Changes in inflammatory signaling pathway proteins in PM cells (**A**) and quantitative analysis of corresponding proteins (**B**–**D**). Changes in RNA levels of PPARγ (**E**) and NF-κB (**F**) in PM cells in Transwell chambers. The protein expression of IRS1, PI3K, and β-actin in 3T3-L1, which was extracted from 3T3-L1 and Raw264.7, co-cultured in the Transwell plate (**G**–**I**). Con: control group; model: mature adipocyte supernatant was incubated with 100 μM PA. #### *p* < 0.0001 significantly compared with Con group; ### *p* < 0.001 significantly compared with Con group; ## *p* < 0.01 significantly compared with Con group; # *p* < 0.05 significantly compared with Con group; *** *p* < 0.001 significantly compared with model group; ** *p* < 0.01 significantly compared with model group; * *p* < 0.05 significantly compared with model group.

The Western blot analysis was performed on the ADSC-PM co-culture model to detect the changes in inflammatory signaling pathway-related proteins in macrophages. Figure 8 shows the Western blot results (A) and protein level quantitative analysis (B). Compared with the Con group, the 100 μM PA group significantly reduced the expression of PPARγ in macrophages (*p* < 0.01); the 100 μM PA + ROS group and 100 μM PA + Rb1 group increased the expression of PPARγ 2.1-fold and 1.3-fold (*p* < 0.001, *p* < 0.05). There was no difference between the 100 μM PA + GW9662 + Rb1 group and the model group. PPARγ expression was increased 1.2-fold by PMA + Rb1 compared with the 100 μM group.

Compared with the Con group, the 100 μM PA group could promote the activation of NF-κB, thereby upregulating the expression of p-NF-κB nearly 2.1-fold (*p* < 0.0001). The 100 μM PA + ROS group and the 100 μM PA + Rb1 group could inhibit the activation of NF-κB, thus reducing the expression of p-NF-κB (*p* < 0.001, *p* < 0.01). There was no difference between 100 μM PA + GW9662 + Rb1 and 100 μM PA. Compared with the 100 μM PA group, the PMA + Rb1 group inhibited the activation of NF-κB and upregulated the expression of p-NF-κB (*p* < 0.05).

Compared with the Con group, the 100 μM PA could significantly reduce the expression of IKBα (*p* < 0.05), 100 μM PA + ROS and 100 μM PA + Rb1 could significantly increase the expression of IKBα compared with the 100 μM PA group (*p* < 0.0001, *p* < 0.001), but there was no difference between the 100 μM PA + GW9662 + Rb1 and the 100 μM PA group. Compared with the 100 μM PA group, the expression of IKBα in the PMA + Rb1 group was significantly upregulated (*p* < 0.05).

In order to explore the specific regulatory pathway and mechanism of obesity-induced insulin resistance on macrophages, RNA in the upper chamber was collected in the ADSC-PM co-culture model for PCR detection, and the changes in RNA level in the PPARγ/NF-κB pathway were mainly detected. As shown in Figure 8E, compared with the Con group, the RNA level of PPARγ in the 100 μM PA group decreased to 0.45 times (*p* < 0.01). After ROS and Rb1 were given, the RNA level of PPARγ in the 100 μM PA group was significantly increased (*p* < 0.01, *p* < 0.05). However, when macrophages were treated with GW9662 in advance, the rising effect of Rb1 administration was eliminated. As shown in Figure 8F, the RNA level of NF-κB in the 100 μM PA group was slightly decreased compared with the Con group (*p* < 0.05), and the RNA level was increased after ROS and Rb1 treatment. However, when macrophages were treated with GW9662 in advance, the increasing effect of drug administration was eliminated, and there was no difference between the Con group and the model group.

Likewise, after co-culture RAW264.7 and 3T3-L1, the IRS1 and PI3K of 3T3-L1 were all decreased compared with those of Con (*p* < 0.01, *p* < 0.001), while ROS and Rb1 could reverse the expression of IRS1 (*p* < 0.001, *p* < 0.01) and PI3K (Figure 8G–I).

## 3. Discussion

Potential targets were identified through network pharmacological studies, among which EGFR, JUN, VEGFA, STAT3, HSP90AA1, and PTGS2 showed a high degree of correlation and were the key nodes in this network. Thus, Rb1 may be related to the above targets in alleviating insulin resistance in obesity.

The biological function of PPARγ (peroxisome proliferator-activated receptor) is also related to its structure. PPARγ usually binds to RXRα as a heterodimer. When stimulated by ligands, PPARγ and RXRα reform activated complexes with co-activating protein PPARγ specific reactive element (PPRE) [17,18]. Another way of structurally reacting PPARγ is ligand direct PPARγ binds to NF-κB and thus inhibits the binding of NF-κB to other target genes. PTGS2 (COX-2) is the downstream protein associated with NF-κB, so its related PPARγ pathway was selected for molecular docking verification [19,20]. The results showed that the binding energy of PPARγ-Rb1 was −6.8 kcal/mol, which can be spontaneously bound.

The primary culture cell is cultured immediately after being removed from the body. Thus, the cells keep the basic properties of the original cells, and the models are often closer to the body’s micro-environment. Studies have shown that vascular endothelial growth factor (VEGF) is an active regulator of brain-derived neurotrophic factor (BDNF) production in diabetic patients after extraction of primary retinal Müller cells [21]. Sami [22] demonstrated that hepatocyte growth factor (HGF)-based ADSC therapy may be useful in the treatment of liver fibrosis in diabetic patients. Thus, the primary cell co-culture model was used as a whole model to simulate the body tissue or organ, and the stress response of macrophages was studied under the condition of adipose tissue obesity. In addition, our previous studies verified that the activation of PPARγ could alleviate obese insulin resistance with the IRS-1 insulin metabolic pathway in vivo and in vitro [23,24,25]. In addition, a few reports show that Rb1 could alleviate insulin resistance in mechanisms. Zou H [15] found that Rb1 likely modulated the gut microbiota and intestinal free fatty acid profiles. Researchers found that Rb1 can alter the amino acids associated with diabetes, such as branched-chain amino acids, tryptophan, and alanine [26]. Furthermore, our results showed that the molecular mechanism of Rb1 inhibiting macrophage activation was through the activation of PPARγ in the obesity microenvironment. By using PPARγ agonist rosiglitazone, PPARγ inhibitor GW9662, or NF-κB agonist PMA the expression of NF-κB and macrophage activation-related pathway proteins were blocked, and the secretion and release of inflammatory factors were affected. Therefore, PPARγ is the potential target of this inflammatory regulation. When the expression of PPARγ protein in the inflammatory signaling pathway is inhibited, IκBα is degraded and cannot bind to NF-κB. Thus, NF-κB is activated and transcribed into the nucleus, where inflammatory factors such as TNF-α and MCP-1 are released, exacerbating the inflammatory response. In addition, when the expression of IRS1 protein in hypertrophic adipocytes is inhibited, it could reduce insulin sensitivity and aggravate IR [27]. However, Rb1 could upregulate the expression of IRS1 and PI3K protein and indirectly affect the insulin signal pathway, which had a similar effect to ROS. It can be further demonstrated that Rb1 might alleviate insulin resistance by PPARγ in obese insulin resistance. Moreover, other predicted targets have been focused on in further experiments. For instance, Emanuele [28] concluded from single-cell transcriptomics that EGFR is the high central gene of T2D. Yaping [29] found that VEGFA was highly upregulated in the T2D retina; it is a new potential therapeutic target and biomarker for proliferative diabetic retinopathy treatment and diagnosis [30]. These have been predicted in T2D PPI network related as crucial targets. These targets or pathways could be concerned further about the correlation with PPARγ.

In conclusion, Rb1 could affect the secretion and release of inflammatory factors through the important target of PPARγ, regulate the activation of macrophages, and further inhibit the occurrence of insulin resistance in obesity. PPARγ might be the potential target for Rb1 in obesity-related insulin resistance, which would be a therapy for early T2D treatment.

## 4. Materials and Methods

### 4.1. Materials

Compound Rb1 (purity: 98%) was obtained from the Shanghai yuan ye Bio-Technology. Insulin (Ins), 1-methyl-3-isobutylxanthine (IBMX), dexamethasone (Dex), and palmitic acid (PA) were purchased from Sigma (USA). Dulbecco’s modified eagle’s medium (DMEM) and penicillin-streptomycin (PS) were obtained from the Hyclone (Logan, UT, USA). NF-κB, p-NF-κB, and IκBα antibodies were purchased by Abcam (USA). PPARγ β-actin antibodies were purchased by Santa Cruz. TG kit was obtained from Zhejiang Dongou Diagnostic Products Co., Ltd. TNF-α, MCP-1, IL-1β, IL-10, and ARG1 ELISA kits were obtained from BioLegend.

### 4.2. The Putative Targets of Rb1

Previously, we used the PubChem database (https://pubchem.ncbi.nlm.nih.gov/, accessed on 11 January 2023) to find the canonical SMILES of Rb1, with the help of the SwissTargetPrediction (http://www.SwissTargetprediction.ch/, accessed on 11 January 2023) database to predict the corresponding potential targets.

### 4.3. Acquisition of T2D Disease-Related Targets and Drug-Disease Common Targets

Using the GeneCards database (https://www.genecards.org/, accessed on 11 January 2023), OMIM (http://omim.org/, accessed on 11 January 2023), DisGeNET (https://www.disgenet.org/, accessed on 11 January 2023), CTD (http://ctdbase.org/, accessed on 11 January 2023), and DrugBank (https://go.drugbank.com/, accessed on 11 January 2023) databases, and “Type 2 diabetes” was used as the search entry to obtain the relevant targets. The active component targets of Rb1 and T2D disease targets obtained in item 2.2 were imported into Venny 2.1. The line tool was used to draw the Venny diagram to obtain the common targets of Rb1-T2D.

### 4.4. Construction and Visualization of Protein-Protein Interaction (PPI) Network

The common targets of Rb1-T2D were imported into the STRING database (https://string-db.org/, accessed on 11 January 2023) for analysis, and a protein–protein interaction network was constructed. In the network, nodes represented molecular species, and edges connected between nodes represented intermolecular interactions. The node degree value represents the number of edges connected to the node. The larger the value, the more nodes are connected to the node, indicating that the node is more important in the network and plays the role of a hub in the whole network, which may be the key target point.

### 4.5. GO Biological Function and KEGG Pathway Enrichment Analysis

Based on the DAVID 6.8 database (https://david.Ncifcrf.gov/, accessed on 11 January 2023) about intersection targets for Gene Ontology (GO) functional annotation and Kyoto Encyclopedia Of Genes and Genomes (KEGG) pathway enrichment analysis. Using microscopic (http://www.Bioinformatics.com.cn/, accessed on 11 January 2023) online platform drawing tools to go online and KEGG pathway enrichment results in visualization analysis.

### 4.6. Molecular Modeling

The PDB file of PPARγ from the RSCB PDB database (https://www.rcsb.org/, accessed on 11 January 2023) was downloaded, and then the ligand and non-protein molecules (such as water molecules) were removed from the target protein and saved as a PDB file. The mol.2 format file of Rb1 from the TCMSP database (https://old.tcmsp-e.com/tcmsp.php, accessed on 11 January 2023) was downloaded. The software was used to upload the protein file after water hydrogenation, and the software was used to convert it into a *pdbqt format file. Finally, Vina was used for docking. If the binding energy is less than 0, it indicates that the ligand and receptor can spontaneously bind. Here, the binding energy ≤ 5.0 kJ/mol is considered to be well bound.

### 4.7. Extraction and Isolation of ADSCs

C57BL/6J male mice of 3–4 weeks of age were sacrificed and sterilized, placed in the ultra-clean table, and then the epididymal fat was removed and placed in precooled PBS buffer. Next, it was cut with ophthalmic scissors and transferred to 50 mL EP tube. An appropriate amount of type I collagenase was added and shaken at 37 °C water bath for 1 h, filtered through 200 mesh screen to remove residual residue, then transferred to a new 50 mL EP tube, left for 15–30 min, centrifuged, supernatant removed, washed with PBS, centrifuged to discard supernatant. After resuspending, the precipitate was inoculated in T25 flask and cultured in 5% CO_2_ incubator at 37 °C. After 3 days, the first liquid was changed, and after 5–7 days, the first passage was carried out at a ratio of 1:2.

### 4.8. Differentiation Is Induced by ADSC

ADSCs were seeded in 6-well plates at 1 × 10^5^/well. When the growth reached contact inhibition, the solution was changed. They were induced by M1 (DMEM medium containing 10% fetal bovine serum, 0.5 mM IBMX, 1 μM DEX, and 10 μg/mL insulin) for two days. Then they were induced by M2 (DMEM medium containing 10% fetal bovine serum and 10 μg/mL insulin) for two days. Furthermore, they changed the solution again to DMEM medium containing 10% fetal bovine serum for two days and then every two days until differentiation was complete. The differentiation cycle was usually 8–12 days. After the cells were induced to differentiate and mature, the adipogenic ability was observed by oil Red O staining.

### 4.9. PM Extraction and Separation

C57BL/6J male mice aged 6–8 weeks were sacrificed and disinfected, and the abdominal epidermis was cut off with ophthalmic scissors. After disinfection, 3–5 mL of precooled culture medium was injected intraperitoneally. The culture medium containing macrophages was absorbed, transferred to a new 15 mL EP tube, placed on ice, centrifuged at 1000 rpm for 10 min, washed twice with PBS, discarded by centrifugation, added to the culture medium, and resuspended on a 24-well plate or a 96-well plate. After 2 h incubation in the incubator environment, the non-adherent cells in the culture flask were slowly washed with the culture medium. The medium was then replaced with a normal medium.

### 4.10. Identification of PM

The cells were washed with PBS three times, 3 min each time, and incubated with endogenous peroxidase blocker for 10 min at room temperature. After immersion in PBS 3 times, each time for 3 min, a non-immune serum of animals was added and incubated for 10 min at room temperature, and the blocking solution was blotted with absorbent paper. A sufficient amount of primary antibody (CD68) diluted to an appropriate concentration was added to each section by drop, and the samples were incubated at 4 °C overnight or 1–2 h at room temperature in a wet box. The remaining PBS was blotted with absorbent paper, and a sufficient amount of diluted secondary antibody (biotin-labeled sheep anti-rabbit) was added to the sample and incubated for 10 min at room temperature. The remaining PBS was blotted with filter paper, and Streptomyces antibiotic protein-peroxidase was added by drop and incubated at room temperature for 10 min. The remaining PBS was blotted with absorbent paper 3 times, 3 min each time. After the DAB color development solution was configured and added, the dye was rinsed with distilled water, and the staining degree was observed under a microscope. Hematoxylin was stained for 2 min and rinsed with distilled water.

### 4.11. CCK 8 Experiments

The cells to be tested were cultured in 96-well plates. After 24 h, the supernatant was discarded, 100 μL medium and 10 μL CCK8 solution were added to each well, and the cells were cultured in a carbon dioxide incubator at 37 °C for 1–4 h. The absorbance value was measured at 450 nm wavelength with a microplate analyzer, and the survival rate of the cells could be calculated according to the absorbance value.

### 4.12. Establishment of Cell Hypertrophy Model

After ADSCs were differentiated into adipogenic adipocytes, they were seeded in 96-well plates. When the cell density reached 70–80%, the CCK8 method was used to detect the effects of different palmitic acid concentrations on cell viability after 24 h of incubation. In addition, the influence of this range of concentration on cell viability after 24 h incubation of PM was detected to screen the appropriate concentration for subsequent modeling experiments.

### 4.13. Oil Red O Staining

ADSCs were seeded on 6-well plates. After differentiation or induction of differentiation, different concentrations of drugs were added and treated for 24 h; the supernatant was discarded, and the supernatant was gently rinsed with PBS 3 times. An appropriate amount of 4% paraformaldehyde was added to each well and fixed for 20 min. The well plate was rinsed with PBS, and each well was cleaned three times. An appropriate amount of diluted oil red O supernatant was added to each well and incubated overnight. Each well was rinsed three times with distilled water and photographed under a microscope.

### 4.14. Determination of TG Content

After oil red O staining, 1 mL isopropanol was added to each well to resuspend the precipitation, and the solution was collected after the precipitation was fully dissolved. The instructions for the TG kit were followed, and the TG content was calculated.

### 4.15. Cell Co-Culture and Migration Efficiency Detection

Co-culture system: The lower layer of the Transwell plate was inoculated with ADSCs at a concentration of 1 × 10^5^ (600 μL) per well. After the cell differentiation was induced, the upper chamber was inoculated with PM cells at a density of 1.2 × 10^5^ (200 μL) per well. When the cell density reached 80%, the lower layer was stimulated with PA, and the upper layer was treated with Rb1 for 24 h. At the same time, macrophages were given rosiglitazone, GW9662, or NF-κB agonist PMA. At the end of the culture, 400 μL of 4% paraformaldehyde was added to each well of the lower layer, and the chamber was immersed in it and fixed for 20 min. Then, 400 μL of crystal violet dye was added to each well, and the chamber was stained for 30 min. Macrophages in the upper layer of the chamber were wiped with cotton swabs, washed with distilled water, and washed three times in each well. 

### 4.16. Detection of Cytokines in Cell Supernatant

After modeling, the lower supernatant of the Transwell plate was collected in a 1.5 mL EP tube, centrifuged at 3500 rpm at 4 °C for 15 min, cell debris was discarded, and the supernatant was slowly collected in a new 1.5 mL EP tube. The liquid in the EP tube was mixed evenly, and the appropriate ELISA kit was selected to operate according to its instructions, including macrophage M1 cytokines TNFα and IL-1β, macrophage M2 cytokines ARG1 and IL-10, and chemokine MCP-1. The absorbance value was obtained at the corresponding wavelength. The cytokine content was calculated according to the OD value.

### 4.17. Immunofluorescence

The hypertrophic adipocyte model was established by 3T3-L1, RAW264.7 were incubated with the supernatant of the culture medium, and ginsenoside Rb1 was diluted to 20 μM and 30 μM with a supernatant medium. After the modeling, RAW264.7 was incubated with a primary antibody mixture of CD11c (ab11029) and CD206 (ab64693) at 4 °C overnight, followed by incubation of secondary antibodies, including anti-mouse IgG FITC and anti-rabbit IgG PE for 1.5 h at room temperature. Then they were stained with 4’,6-diamidino-2-phenylindole for 10 min and viewed using a fluorescence microscope.

### 4.18. Western Blot Analysis

Proteins were extracted from the cell, and protein concentration was measured with a BCA kit. Primary antibodies against PPARγ (sc7273), p-NF-κB p65 (ab28856), NF-κB p65 (ab16502), IκB-α (ab7217), IRS1 (ab52167), PI3K (sc375534), and β-actin (sc47778) were diluted 1:1000 in PBST buffer. The corresponding secondary antibodies involving goat-anti-mouse and goat-anti-rabbit (Proteintech) were diluted 1:5000 and 1:20,000.

### 4.19. PCR

After 24 h of drug treatment, the Transwell chamber was washed three times with PBS. An amount of 250 μL Trizol was added to each well and the Trizol was allowed to stand at room temperature for 5 min to fully lysate. Then the supernatant was centrifuged at 12,000 rpm for 5 min at 4 °C, and the supernatant was transferred to a new 1.5 mL EP tube. A certain amount of chloroform was added to 200 μL chloroform/mL Trizol, shaken, and mixed until emulsion, placed at room temperature for 15 min, then centrifuged at 12,000× *g* at 4 °C for 15 min. The upper aqueous phase was absorbed into another 1.5 mL EP tube, a certain amount of isopropanol was added to the ratio of 500 μL isopropanol/mL Trizol, shaken up and down, mixed well, and left at room temperature for 10 min. At this time, the RNA was submerged in the bottom of the tube. Then a certain amount of 75% ethanol was added for gentle shaking. The RNA was centrifuged at 4 °C for 10 min at 12,000× *g*, and the supernatant was carefully discarded. An amount of 20 μL DEPC water was added, the bottom of the tube was flicked to dissolve the RNA precipitate fully, and the RNA concentration was measured on a microplate reader.

### 4.20. Statistical Analysis

The data were presented as means ± SEM. All data were analyzed using a two-tailed t-test or one-way analysis of variance (ANOVA) between two groups. All the analyses were carried out using GraphPad Prism 8.0. *p* < 0.05 and *p* < 0.01 were considered statistically different and significantly different, respectively.

## Data Availability

Not applicable.

## Sample Availability

Samples of the compounds are not available from the authors.
